# RETRACTION: Removal of Pb (II) Ions from Aqueous Solutions by *Cladophora rivularis* (Linnaeus) Hoek

**DOI:** 10.1155/tswj/9813586

**Published:** 2025-09-12

**Authors:** The Scientific World Journal

RETRACTION: N. Jafari and Z. Senobari. “Removal of Pb (II) ions from aqueous solutions by Cladophora rivularis (Linnaeus) Hoek,” *The Scientific World Journal* 2012, no. 1 (2012): 793606, https://doi.org/10.1100/2012/793606.

The above article, published online on 3 May 2012 in Wiley Online Library (https://wileyonlinelibrary.com/), has been retracted by agreement between the Associate Editor and John Wiley & Sons Ltd. The retraction has been agreed following an investigation which revealed substantial overlap between this article and another article by the same author [1]. There are also discrepancies in the data between both articles, specifically Table 1 in this article and Table 2 in [1].

The authors did not respond to the retraction.
